# Effects of exercise on depressive symptoms in adults with arthritis and other rheumatic disease: a systematic review of meta-analyses

**DOI:** 10.1186/1471-2474-15-121

**Published:** 2014-04-07

**Authors:** George A Kelley, Kristi S Kelley

**Affiliations:** 1 Department of Biostatistics, Robert C. Byrd Health Sciences Center, West Virginia University, PO Box 9190, 26506-9190 Morgantown, WV, USA

## Abstract

**Background:**

Depression is a major public health problem among adults with arthritis and other rheumatic disease. The purpose of this study was to conduct a systematic review of previous meta-analyses addressing the effects of exercise (aerobic, strength or both) on depressive symptoms in adults with osteoarthritis, rheumatoid arthritis, fibromyalgia and systemic lupus erythematous.

**Methods:**

Previous meta-analyses of randomized controlled trials were included by searching nine electronic databases and cross-referencing. Methodological quality was assessed using the Assessment of Multiple Systematic Reviews (AMSTAR) Instrument. Random-effects models that included the standardized mean difference (SMD) and 95% confidence intervals (CIs) were reported. The alpha value for statistical significance was set at p ≤ 0.05. The U_3_ index, number needed to treat (NNT) and number of US people who could benefit were also calculated.

**Results:**

Of the 95 citations initially identified, two aggregate data meta-analyses representing 6 and 19 effect sizes in as many as 870 fibromyalgia participants were included. Methodological quality was 91% and 82%, respectively. Exercise minus control group reductions in depressive symptoms were found for both meta-analyses (SMD, -0.61, 95% CI, -0.99 to -0.23, p = 0.002; SMD, -0.32, 95% CI, -0.53 to -0.12, p = 0.002). Percentile improvements (U_3_) were equivalent to 22.9 and 12.6. The number needed to treat was 6 and 9 with an estimated 0.83 and 0.56 million US people with fibromyalgia potentially benefitting.

**Conclusions:**

Exercise improves depressive symptoms in adults with fibromyalgia. However, a need exists for additional meta-analytic work on this topic.

## Background

Arthritis is a broad term used to describe more than 100 rheumatic diseases and conditions that affect joints as well as the surrounding tissues around joints [1]. The most common form of disability in the United States (US), arthritis affects all racial and ethnic groups and is more common in women than men [1]. Based on 2007–2009 data, the prevalence of doctor-diagnosed arthritis in the US was reported to be 50 million, or about 20%, of all adults [2]. In terms of costs, an increase of 41.8 billion dollars in total costs (from 86.2 to 128 billion dollars) was reported between 1997 and 2003 in the US [3].

Four common types of arthritis and other rheumatic diseases are osteoarthritis, rheumatoid arthritis, fibromyalgia and systemic lupus erythematous. More specifically, the prevalence of osteoarthritis, rheumatoid arthritis, fibromyalgia and systemic lupus erythematous have been estimated to be 27 million [4], 1.5 million [5], 5 million [4], and 161,000 [4], respectively. A common problem among adults with arthritis is depression. For example, a recent study that included 1,793 US adults 45 years of age and older with arthritis found that 18% had depression while only slightly more than half (51.3%) sought help for their depression [6].

One potential treatment option for adults with arthritis and depression is exercise, a low-cost nonpharmacologic intervention that is available to the vast majority of the general population. Systematic reviews with meta-analysis, a quantitative approach for combining the results of different studies on the same topic [7], are considered by many to be the most important type of evidence for determining the efficacy and effectiveness of various treatments on selected outcomes [8,9]. Unfortunately, with the proliferation of systematic reviews on the same topic, it becomes difficult to make informed decisions regarding the effects of various interventions on selected outcomes. For example, a recent systematic review identified 33 previous meta-analyses examining the effects of exercise on blood pressure [10]. Given the proliferation of systematic reviews, with or without meta-analysis on the same topic, a need now exists to systematically review these previous reviews in order to provide decision-makers and practitioners with the information they need to make evidence-based decisions regarding the efficacy and effectiveness of various interventions on selected outcomes as well as provide researchers with direction for future research [11]. Given the former, the purpose of the current study was to conduct a systematic review of previous meta-analyses addressing the effects of exercise (aerobic, strength training or both) on depressive symptoms in adults with osteoarthritis, rheumatoid arthritis, fibromyalgia or systemic lupus erythematous.

## Methods

### Study eligibility

The *a priori* inclusion criteria for this study were as follows: (1) previous systematic reviews with meta-analysis of randomized controlled trials or data reported separately for randomized controlled trials if the meta-analysis included other study designs, (2) adults 18 years of age and older with osteoarthritis, rheumatoid arthritis, fibromyalgia or systemic lupus erythematous, as defined by the inclusion criteria of the authors of the original meta-analyses, (3) aerobic and/or strength training intervention(s) lasting an average of at least 4 weeks, (4) published and unpublished (dissertations and master’s theses) studies in any language, (5) exercise minus control group difference in depressive symptoms as a primary outcome in the original meta-analysis and reported as the standardized mean difference (SMD). Meta-analyses were limited to randomized controlled trials because they are the only way to control for unknown confounders as well as the fact that nonrandomized controlled trials tend to overestimate the effects of treatment in healthcare interventions [12,13]. In addition, meta-analyses in which the focus was on acute studies, for example studies in which participants would perform one or more bouts of exercise and then immediately be assessed for depressive symptoms, were avoided. Given the different instruments used to assess depressive symptoms, the inclusion of meta-analyses were limited to those in which the SMD was reported. Any studies that did not meet all of the above criteria were excluded. Ineligible studies were excluded based on at least one of the following: (1) inappropriate population (for example, children), (2) inappropriate intervention (for example, pharmacologic), (3) inappropriate comparison (for example, exercise versus pharmacologic), (4) inappropriate outcome (for example, anxiety), (5) inappropriate study type (for example, meta-analysis that included non-randomized controlled trials, systematic review without meta-analysis).

### Data sources

Using the graphical-user interfaces for each database, the following electronic sources were searched from their inception forward: (1) PubMed (1966 to July 4, 2013), (2) Sport Discus (1975 to July 4, 2013), (3) Web of Science (1955 to July 4, 2013), (4) Scopus (1823 to July 4, 2013), (5) Proquest (1861 to July 4, 2013), (6) Cochrane Database of Systematic Reviews (1996 to July 4, 2013), (7) Physiotherapy Evidence Database [(PEDRO) (1929 to July 5, 2013)], (8) Database of Abstract of Reviews of Effects (DARE) (1991 to July 5, 2013), (9) Health Evidence Canada (HEC) (1985 to July 5, 2013). Scopus was included in our database searches because it has been reported to provide coverage of Embase, a database that was not available to us [14]. While the specific search strategies varied depending on the database searched, key terms or forms of key terms included exercise, physical activity, physical fitness, arthritis, fibromyalgia, lupus, randomized, depression, systematic review and meta-analysis. A copy of the search strategies used for each database can be found in Additional file 1. After removing duplicates, the overall precision of the searches was calculated by dividing the number of studies included by the total number of studies screened [15]. The number needed to read (NNR) was then calculated as the inverse of the precision [15]. In addition to electronic database searches, cross-referencing for potentially eligible meta-analyses from retrieved reviews was also conducted. All studies were stored in Reference Manager, version 12.0 [16].

### Study selection

All studies were selected by both authors, independent of each other. They then met and reviewed their selections for agreement. Any disagreements were resolved by consensus.

### Data abstraction

Prior to data abstraction, coding sheets were developed in Microsoft Excel 2010 [17]. The coding sheets could hold up to 193 items from each included meta-analysis. Both authors coded all studies independent of each other. Upon completion of coding, all coding sheets were merged into one common codebook and reviewed by both authors for correctness. Disagreements were resolved by consensus. Using Cohen’s kappa statistic [18], the overall agreement rate prior to correcting discrepancies was *k* = 0.88, considered to be “almost perfect” [19].

### Methodological quality

Methodological quality for each included meta-analysis was assessed using the Assessment of Multiple Systematic Reviews (AMSTAR) Instrument [20-23]. AMSTAR was chosen over other instruments [24,25] because of its reported inter-rater reliability (*k* = 0.70), construct validity (intra-class correlation coefficient = 0.84) and feasibility (average of 15 minutes per study to complete) [22]. The 11-item questionnaire is designed to elicit responses of “Yes”, “No”, “Can’t Answer”, or “Not Applicable”. The response “Can’t Answer” is chosen when an item is relevant but not described. The response “Not Applicable” is chosen when an item is not relevant (for example, meta-analysis of data not possible) [20-23]. For consistency when summing responses, the following question was modified from “Was the status of publication (i.e. grey literature) used as an inclusion criterion?” to “Was the status of publication (i.e. grey literature) as an inclusion criterion avoided?” In addition, we considered the question regarding conflict of interest as adequately met if the authors of the systematic review provided a statement on conflict of interest versus the reporting of conflict of interest by both the authors of the systematic review and all the original studies included in the meta-analysis. Both authors, independent of each other, assessed methodological quality. They then met and reviewed every item for agreement. Disagreements were resolved by consensus. The overall agreement rate prior to correcting discrepancies was *k* = 0.94, considered to be “almost perfect” [19].

### Data synthesis

The main results from each meta-analysis were extracted *a priori*[7] with a focus on random-effects models because they incorporate between-study heterogeneity into the model [26,27]. The SMD, 95% confidence intervals (CIs) and associated z and alpha value for z were abstracted or calculated if sufficient data were available to do so. Standardized mean differences were classified as trivial (<0.20), small (0.20 to 0.49), medium (0.50 to 0.79) or large (>0.80) [28]. Two-tailed alpha values ≤ 0.05 along with non-overlapping 95% CIs were considered as statistically significant. The Q statistic, a measure of heterogeneity, was also extracted for each outcome. An alpha value ≤ 0.10 was considered to represent statistically significant heterogeneity [29]. Because of issues surrounding the power of the Q statistic, the *I*^*2*^ statistic was also reported if it was provided in the meta-analysis or calculated if sufficient data existed to do so [29]. Values were considered to be representative of low (0 to 25%), moderate (25 to 50%), large (50 to 75%) or very large (>75%) inconsistency [29]. In addition to Q and *I*^*2*^*,* tau-squared (τ^2^) was also reported or calculated if sufficient data were available. An *a priori* decision was made to not pool results from the different meta-analysis because of the expectation that many of the same studies would be included in the different meta-analyses, thus violating the assumption of independence. *Post hoc*, a decision was made to pool the results of one included meta-analysis [30] that reported separate results for studies that met the American College of Sports Medicine (ACSM) Guidelines for aerobic fitness [31,32] (4 studies), those that did not meet the recommendation (1 study), and those in which strength training was performed (1 study). The rationale for this decision was based on the desire to examine exercise in a broader context. However, results were also reported separately as was done in the original meta-analysis [30].

Since it was assumed that none of the eligible meta-analyses would include 95% prediction intervals (PIs), these were calculated if the findings were statistically significant and the results from each study included in each meta-analysis were provided [33-35]. Prediction intervals are used to estimate the treatment effect in a new trial [33-35] and may be more appropriate in decision analysis [36].

In order to enhance practical application, the number-needed-to treat (NNT) was calculated for any overall findings that were reported as statistically significant. This was accomplished using the approach suggested by the Cochrane Collaboration and was based on a control group risk of 30% [8]. Since both included meta-analyses were limited to depressive symptoms in those with fibromyalgia [30,37], a *post hoc* decision was made to provide a gross estimate, based on the NNT results, of the number of US people with fibromyalgia who could improve their depressive symptoms by starting and maintaining a regular exercise program. Estimates were based on previous research showing that approximately 5 million people in the US have fibromyalgia [4]. In addition, Cohen’s U_3_ index was used to determine the percentile gain in the intervention group [38]. For example, a SMD of 0.50 suggests that on average, a person in the exercise group would be at the 69^th^ percentile in terms of improving their depressive symptoms. This translates into being 19 percentiles higher than the control group [39].

If not already provided and if sufficient data were available to do so, small-study effects (for example, publication bias) were assessed quantitatively using the regression-intercept approach of Egger et al. [40]. One-tailed alpha values ≤ 0.05 for *t* were considered to be representative of statistically significant small-study effects. In addition, influence analysis was conducted with each SMD deleted from the model once. Cumulative meta-analysis, ranked by year, was also conducted to examine results over time [41]. In addition, since one meta-analysis [37] included studies in which there were active control groups (hot packs, stretching, etc.), we used a two-category, mixed-effects ANOVA model to compare for statistically significant differences between active control groups versus all other included controls (usual care and attention control). A between group alpha level ≤ 0.05 was considered to be statistically significant.

Negative SMDs were indicative of benefit, i.e., decreases in depressive symptoms. Analyses were carried out using Comprehensive Meta-Analysis (version 2.2) [42] and Microsoft Excel 2010 [17].

## Results

### Characteristics of included meta-analyses

**Figure 1:**
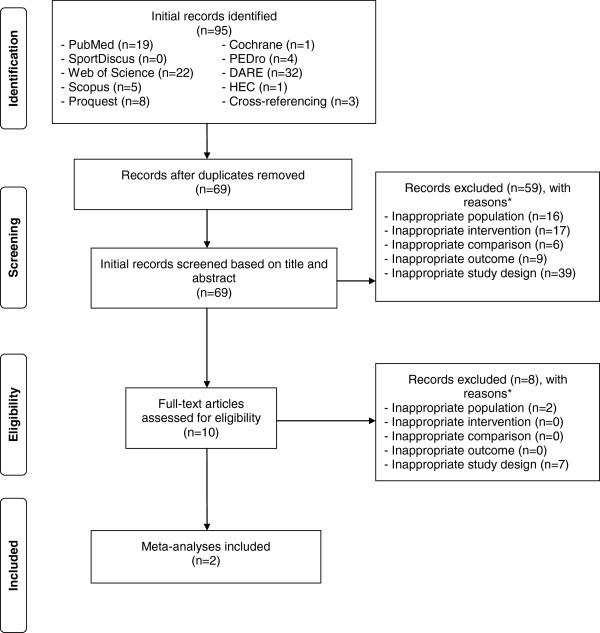
**Flow diagram for the selection of studies.** *, number of reasons exceeds the number of studies because some studies were excluded for more than one reason.

Of the 95 citations initially identified, 69 (72.6%) remained after removing duplicates. Of the 69 articles that were screened, two aggregate data meta-analyses, both in participants with fibromyalgia, met the criteria for inclusion [30,37]. Post hoc, one study that was initially included was removed because it was not focused specifically on the effects of exercise on depressive symptoms in adults with a specific type of arthritis or other rheumatic disease, but rather, in adults with a variety of chronic illnesses [43]. The precision of the searches was 0.03 while the NNR was 35. The major reasons for exclusion of ineligible studies were an inappropriate study design (48.4%) followed by an inappropriate intervention (17.9%), population (18.9%), outcome (9.5%) and comparison (6.3%). No meta-analysis was deleted because they did not report their results as a SMD. A flow diagram that depicts the search process can be found in Figure 1 while a list of all excluded studies, including the reasons for exclusion, is shown in Additional file 2. For the two included meta-analyses [30,37], one included studies on aerobic or strength training exercise [30] while the second was limited to aerobic exercise studies but also included studies in which participants performed strength training as long as the number of minutes of strength training did not exceed the number of minutes spent performing aerobic exercise [37]. Both meta-analyses included fibromyalgia participants as defined by the diagnostic criteria of the original studies [30,37]. A general description of the characteristics of each meta-analysis is provided in Table 1.

**Table 1:** General characteristics of included meta-analyses

**Reference**	**Year**	**Studies**	**Participants**	**Interventions**
Busch et al. [30]	2007	6	294 primarily women (154 exercise, 137 control), all with fibromyalgia, 33–50 years of age $$\left(\bar{X}\pm \mathrm{SD},41.1\pm 5.2\right)$$	Supervised/unsupervised aerobic and strength training interventions lasting 6 to 23 weeks $\left(\bar{X}\pm \mathrm{SD},14\pm 7\;\mathrm{weeks}\right)$, frequency (2-5x week), duration (18–90 minutes per session), intensity (aerobic, 40% -75% HRR; strength, 40%-80% 1RM)
Hauser et al. [37]	2010	18	870 primarily women (456 exercise, 414 control), all with fibromyalgia, 35–56 years of age $$\left(\bar{X}\pm \mathrm{SD},46.6\pm 5.2\right)$$	Supervised/unsupervised aerobic exercise lasting 6–26 weeks $\left(\bar{X}\pm \mathrm{SD},13\pm 6\right)$, frequency $\left(1-7x\;\text{week},\bar{X}\pm \mathrm{SD},3\pm 1\right)$, duration $\left(15-45\;\text{minutes}\;\text{per}\;\text{session},\bar{X}\pm \mathrm{SD},31\pm 10\right)$, intensity (40%-85% MHR), compliance $$\left(38\%-92\%,\bar{X}\pm \mathrm{SD},62\pm 19\right)$$

Notes: $\bar{X}\pm \mathrm{SD}$, mean ± standard deviation; HRR, heart rate reserve; RM, repetition maximum, MHR, maximum heart rate; Description of meta-analyses limited to those related to depressive symptoms as the outcome; Data presented limited to that which was reported or could be calculated from reported data.

### Methodological quality

Item by item results for each meta-analysis using the AMSTAR instrument is shown in Additional file 3. The meta-analysis by Busch et al. [30] satisfied 10 of the 11 (91%) of the AMSTAR criteria while the study by Hauser et al. [37] met 9 of the 11 criteria (82%). One meta-analysis was judged as (1) not avoiding the status of publication as an inclusion criterion and (2) not providing a list of excluded studies [37].

### Data Synthesis

The overall results for both included systematic reviews with meta-analysis are shown in Table 2. As can be seen, SMD reductions in depressive symptoms included non-overlapping confidence intervals for both with one meta-analysis yielding a small SMD [37] and one yielding a medium SMD [30]. While the overall SMD was approximately twice as large for the Busch et al. meta-analysis [30], the between-meta-analysis 95% CIs for both were overlapping, suggesting no statistically significant difference between the two studies [30,37]. In addition, the pooled SMD for the Busch et al. meta-analysis was the result of the current investigative team combining the results from those studies meeting the ACSM guidelines for aerobic exercise with the one strength training study and one aerobic study not meeting the ACSM recommendations, as reported by the authors [30]. A statistically significant and a large amount of heterogeneity were found for both meta-analyses as well as overlapping 95% PIs [30,37]. Data for the NNT, number who could benefit and percentile improvement are shown in Table 3. No small-study effects were observed for the overall meta-analysis results of Busch et al. [30] (β_0,_ -3.8, 95% CI, -10.9 to 3.3, p = 0.11) while statistically significant small study effects were observed for the Hauser et al. [37] meta-analysis (β_0,_ -2.4, 95% CI, -4.8 to -0.01, p = 0.02). With each study deleted from the overall model once for each meta-analysis [30,37], SMD changes in depressive symptoms remained statistically significant with non-overlapping confidence intervals. Results ranged from -0.49 (95% CI, -0.12 to -0.85, p = 0.009) to -0.72 (95% CI, -0.35 to -1.10, p < 0.0001) in the study by Busch et al. [30], and -0.23 (95% CI, -0.08 to -0.38, p = 0.003) to -0.36 (95% CI, -0.15 to -0.57, p = 0.001) in the study by Hauser et al. [37]. Cumulative meta-analysis, ranked by year, revealed that SMD changes in depressive symptoms have remained statistically significant with non-overlapping confidence intervals since 2001 for the meta-analysis by Busch et al. [30] (range of years, 1996 to 2004) and from 2004 onward in the meta-analysis by Hauser et al. [37] (range of years, 1996 to 2009). No statistically significant difference was found between active control groups and the other types of control groups included in the Hauser et al. meta-analysis (Q_b_ = 1.13, p = 0.29) [37].

**Table 2:** Overall post-treatment standardized mean difference (SMD) effect sizes for depressive symptoms from included meta-analyses

**Reference**	**ES/Participants (No.)**	**SMD (95% CI)**	**Z(p)**	**Q (p)**	***I***^***2 *(%)**^	**τ**^**2**^	**PI (95%)**
Busch et al. (2006) [30]^a^	6/294	**-0.61 (-0.99 to -0.23)**	-3.12(0.002)*	12.0 (0.04)**	58.4	0.13	-1.74, 0.53
Hauser et al. (2010) [37]	19/870	**-0.32 (-0.53 to -0.12)**	-3.13(0.002)*	37.0 (0.005)**	51.4	0.22	-1.32, 0.68

Notes: No, Number; ES, effect size; SMD, standardized mean difference; 95% CI, 95% confidence intervals; Z(p), Z-value and probability value for Z; Q(p), Cochran’s Q statistic and associated alpha (p) value for Q; *I*^*2*^*,* I-squared statistic for heterogeneity; τ^2^, tau-squared; PI, prediction intervals, based on a random-effects model; SMD (95% CI) based on random-effects model; **Boldfaced** values indicate continuous data with non-overlapping confidence intervals; *, statistically significant, p ≤ 0.05; **, statistically significant, p ≤ 0.10; ^a^ , original meta-analysis did not pool results as done here, but rather, reported separate subgroup results for aerobic exercise studies meeting the American College of Sports Medicine recommendations [31,32] (4 studies), those not meeting the recommendations (1 study) and those limited to strength training exercise (1 study).

**Table 3:** NNT, number whose depressive symptoms could benefit from exercise, and percentile improvement

**Reference**	**NNT (95% CI)**	**Number Benefitting (95% CI) (millions)**^**a**^	**U**_**3 **_**Index (95% CI)**^**b **^**(Percentile Improvement)**
Busch et al. (2006) [30]	6 (4, 13)	0.83 (1.25, 0.38)	22.9 (9.1, 33.9)
Hauser et al. (2010) [37]	9 (6, 23)	0.56 (0.83, 0.22)	12.6 (4.8, 20.2)

Notes: NNT, number needed to-treat; 95% CI, 95% confidence intervals; ^a^, gross estimate of the number of US people with fibromyalgia who could improve their depressive symptoms if they started and maintained a regular exercise program. Based on an estimated 5 million US people with fibromyalgia [4] and assuming that none exercise regularly; ^b^, Cohen’s U_3_ Index [38].

Subgroup results were provided for both meta-analyses [30,37]. For the Busch et al. meta-analysis [30], results for depressive symptoms were reported according to studies meeting the ACSM recommendations for aerobic exercise, [30,31] (4 studies), those not meeting the recommendations (1 study) and those limited to strength training exercise (1 study). For those studies meeting the ACSM recommendations, a statistically significant reduction in depressive symptoms was observed (SMD, -0.40, 95% CI, -0.04 to -0.76, p = 0.003). The SMD for the study not meeting the recommendations was -1.22 (95% CI, -1.90 to -0.54) while the SMD for the study in which strength training was performed was -1.14 (95% CI, -2.08 to -0.20). Subgroup analyses in the study by Hauser et al. [37] included results partitioned according to two studies in which low intensity exercise (50% to 60% of maximum heart rate) was compared to moderate intensity exercise (60% to 80% of maximum heart rate) as well as eight studies in which land-based exercise was compared to water-based exercise. The authors reported no statistically significant differences between either low or moderate intensity exercise (SMD, -0.16, 95% CI, -0.67 to 0.13, p = 0.53) or land versus water-based exercise (SMD, -0.44, 95% CI, -0.88 to 0.01, p = 0.05).

## Discussion

### Findings

The purpose of the current study was to conduct a systematic review of previous meta-analyses addressing the effects of exercise (aerobic, strength training or both) in the treatment of depressive symptoms in adults with osteoarthritis, rheumatoid arthritis, fibromyalgia or systemic lupus erythematous. While no meta-analyses were included for participants with osteoarthritis, rheumatoid arthritis or systemic lupus erythematous, two meta-analyses in fibromyalgia participants met the eligibility criteria [30,37]. Generally speaking, it appears that exercise can reduce depressive symptoms in adults with fibromyalgia. This interpretation is supported by (1) the non-overlapping confidence intervals for SMDs, (2) statistical significance of the SMDs, (3) sensitivity of results with each study deleted from the model once, (4) cumulative meta-analysis, (5) low NNT, (6) absolute number of people in the US who might benefit by starting and maintaining an exercise program, (7) percentile improvements as a result of exercise and (8) good overall methodological quality of each meta-analysis as assessed by the AMSTAR instrument. However, for both [30,37], a statistically significant and relatively large amount of heterogeneity was observed as well as overlapping prediction intervals. In addition, small-study effects were found for the Hauser et al. [37] meta-analysis and based on the work of others [44], may have been underpowered for the Busch et al. [30] study. Consequently, the strength of the overall findings may be weakened by these results.

The overall findings of the included meta-analyses compare quite favorably to the effects of pharmacologic interventions on depressive symptoms in adults with fibromyalgia. For example, Hauser et al. [45] conducted a meta-analysis of randomized controlled trials on the effects of antidepressants (tricyclic and tetracyclic antidepressants, selective serotonin reuptake inhibitors, serotonin and noradrenaline reuptake inhibitors, monoamine oxidase inhibitors) on depressed mood in adults with fibromyalgia. Across 10 SMDs that included 887 participants (451 treatment, 436 control) a small SMD improvement of -0.26 (95% CI, -0.39, -0.12, p < 0.001) was reported. However, in contrast to the exercise meta-analyses included in the current study [30,37], no statistically significant heterogeneity was observed (Q = 6.39, p = 0.70, *I*^*2*^ = 0%). Thus, while the effects of antidepressants were generally smaller [45], the results were more consistent than the two exercise meta-analyses included in the current study [30,37]. The former notwithstanding, one should also consider the potential side-effects and costs of any type of pharmacotherapy, including antidepressants.

### Implications for research

The results of the current systematic review of previous meta-analyses have at least eight implications for future research. First, while the overall quality of both meta-analyses was considered to be good, there are several areas that might be improved upon in future meta-analytic work. These include avoiding the use of publication status as an inclusion criterion as well as documenting and providing a list of not only included studies but also excluded studies, including the reasons for exclusion. While there is little doubt in the investigators’ minds regarding the latter recommendation, avoiding the use of publication status as an inclusion criterion could be questioned. For example, van Driel et al. suggested that (1) the difficulty in retrieving unpublished work could lead to selection bias, (2) many unpublished trials are eventually published, (3) the methodological quality of such studies are poorer than those that are published, and (4) the effort and resources required to obtain unpublished work may not be warranted [46].

Second, both included studies were aggregate data meta-analyses [30,37]. While still the most common type of meta-analysis, individual-participant data meta-analyses (IPD) have been suggested to be the gold standard when attempting to quantitatively combine data from different studies on the same topic [47]. Thus, future meta-analysts may want to consider using the IPD approach when addressing the effects of exercise in the treatment of depressive symptoms in adults with arthritis and other rheumatic diseases. However, the use of the IPD approach needs to be considered with respect to the ability to retrieve IPD from investigators as well as the increased costs and time associated with the conduct of such [48].

Third, given the apparent paucity of data available on adverse events and cost-effectiveness in the original studies included in both meta-analyses [30,37], there is a need for future randomized controlled trials to collect and report this information. The inclusion of such information is critical when making decisions regarding which interventions to recommend over others.

Fourth, the dose–response effects of exercise on depressive symptoms in adults with fibromyalgia are still unknown. While the meta-analysis by Hauser et al. found no statistically significant differences between either low or moderate intensity aerobic exercise and land versus water-based exercise [37], future research in this area appears warranted. Greater knowledge of the dose–response effects of exercise on depressive symptoms in adults with fibromyalgia should lead to better treatment in this population.

Fifth, no meta-analysis that was limited to the effects of exercise on depressive symptoms in adults with osteoarthritis, rheumatoid arthritis or systemic lupus erythematous met the eligibility criteria for the current study. Since the effects of exercise on depressive symptoms may vary across different populations, it appears plausible to suggest that future meta-analytic work be limited and focused on these groups. This is of course assuming that previous randomized controlled trials have assessed depressive symptoms in these populations.

Sixth, because neither meta-analysis reported NNT with respect to depressive symptoms [30,37], it is suggested that future meta-analytic work include such. From the investigators’ perspective, the inclusion of such information is important because it provides practically relevant information to decision-makers (practitioners, policy-makers, etc.) regarding the effects of exercise on depressive symptoms in adults with fibromyalgia.

Seventh, given the significant heterogeneity in the included meta-analyses, future meta-analytic research on depressive symptoms in adults with fibromyalgia should try and identify the sources of this heterogeneity. Broadly, this may include such things as participant characteristics (for example, age, gender), intervention characteristics (for example, length, frequency, intensity, duration, mode) and outcome assessment methods (for example, type of instrument used to assess depression). Again, this is of course assuming that sufficient data are available to examine these potential predictors.

Eighth, the majority of the participants that comprised both meta-analyses were women [30,37]. The inclusion of primarily women for the studies nested within each meta-analysis appears plausible given that the prevalence of fibromyalgia is greater in women than men [4]. However, it would appear appropriate to suggest that future research examine the effects of exercise on depressive symptoms in men to ensure that no differences in response exist.

### Implications for practice

The results of the current systematic review of previous meta-analyses have important implications for practice. First, while there was a lack of adverse event and cost-effectiveness data as well as substantial between-study heterogeneity in both meta-analyses [30,37], exercise appears to improve depressive symptoms in adults with fibromyalgia and could be recommended as part of an overall treatment plan that may also include education and/or pharmacotherapy. This exercise recommendation is consistent with previous recommendations on aerobic and strength training for a variety of outcomes in adults with fibromyalgia [49,50]. Second, while the dose–response effects of exercise in the treatment of depressive symptoms in adults with fibromyalgia have not been firmly established [51], it would appear prudent to recommend that practitioners follow the general recommendations described by Skinner [51]. These include exercise programs that (1) minimize any increase in pain, fatigue or other symptoms, (2) begin at a low level and progress gradually, (3) allow for day to day variations based on how the participant feels, (4) improve the physiological and psychological functioning of the participant and (5) promote long-term adherence [51]. More specifically, a combined program of low to moderate intensity aerobic exercise (walking and swimming for example) combined with low to moderate intensity strength training may be better tolerated than high intensity activity [51]. Given the day to day variation in how fibromyalgia participants may feel, intensity may be better monitored using something like rating of perceived exertion scales [52-55] versus a percentage of 1-repetition maximum (strength training) and maximum heart rate, heart rate reserve or percentage of maximum oxygen consumption (aerobic training) [51].

### Strengths and potential limitations of current study

There are at least five strengths of the current study. First, to the best of the authors’ knowledge, this is the first systematic review of previous meta-analyses that has examined the effects of exercise on depressive symptoms in adults with arthritis and other rheumatic disease, an increasingly important approach for addressing the effects of various healthcare interventions and making subsequent decisions regarding such [11]. Second, the additional analyses conducted based on the available data (small-study effects, influence analysis, NNT, etc.), helped strengthen the information from which conclusions could be drawn from both included meta-analyses [30,37]. Third, the calculation and inclusion of PIs for the overall results from each included meta-analysis provides investigators with information that can aid them in planning future randomized controlled trials examining the effects of exercise on depressive symptoms in adults with fibromyalgia. Fourth, the investigative team believes that the calculation of percentile improvements, NNT and gross estimates of the absolute number of adults with fibromyalgia who could improve their depressive symptoms by initiating and maintaining a program of regular exercise enhances the practical applicability and importance of findings. Fifth, while the inclusion of only two meta-analyses may initially appear to be a limitation of the current study, the investigators view this as a strength given that as many as 32 SMDs on depressive symptoms in as many as 870 participants across multiple studies were included. To put this in perspective, the Cochrane Collaboration suggests that the minimum number of studies needed to conduct a meta-analysis is two [8]. Given this line of thinking, it would appear plausible to suggest that the minimum number of meta-analyses that need to be included in a study of systematic reviews with meta-analyses is one.

While there are several strengths of the current study, there are also at least four *potential* limitations. First, the investigative team focused on depressive symptoms [30,37]. While more focused and applicable, other relevant outcomes (anxiety, quality of life, quality of sleep, pain, fatigue, stiffness, physical function) were not captured. Second, the gross population estimates for the number of people in the US with fibromyalgia who could improve their depressive symptoms by beginning and maintaining a regular exercise program assumed that none of those with fibromyalgia in the US exercise on a regular basis. Unfortunately, the investigative team is not aware of any current research on the prevalence of physical activity in US adults with fibromyalgia, and thus, was unable to adjust for such. In addition, it was not possible to adjust for any other potentially confounding factors (for example, age). Therefore, the reported estimates might be inflated. Third, as with any systematic review, many of the biases inherent in both the included meta-analyses as well as the randomized controlled trials that comprised each meta-analysis may have also been present in the current study. Fourth, while results were generalized to both men and women, the majority of participants included in both meta-analyses were women [30,37]. Thus, such a generalization may have been inappropriate.

## Conclusions

The results of the current systematic review of previous meta-analyses suggest that exercise improves depressive symptoms in adults with fibromyalgia. However, a need exists for additional meta-analytic work in this area, including, but not limited to, the inclusion of adults with osteoarthritis, rheumatoid arthritis and systemic lupus erythematous.

## Competing interests

The authors declare that they have no competing interests.

## Authors’ contributions

GAK was responsible for the conception and design, acquisition of data, analysis and interpretation of data, drafting the initial manuscript and revising it critically for important intellectual content. KSK was responsible for the conception and design, acquisition of data, and reviewing all drafts of the manuscript. Both authors read and approved the final manuscript.

## Authors’ information

GAK has more than 20 years of successful experience in the design and conduct of all aspects of meta-analysis, including the effects of chronic exercise in adults with arthritis and other rheumatic disease. KSK has more than 16 years of successful experience in conducting meta-analysis, including the effects of chronic exercise in adults with arthritis and other rheumatic disease.

## Pre-publication history

The pre-publication history for this paper can be accessed here:

http://www.biomedcentral.com/1471-2474/15/121/prepub

## Supplementary Material

Additional file 1 Search strategies for databases searched. Click here for file

Additional file 2 Studies excluded, including reasons for exclusion. Click here for file

Additional file 3 **Item by item results using the AMSTAR assessment instrument [**[20-23]. Click here for file
