# Supplementary material for: Effects of exercise on depressive symptoms in adults with arthritis and other rheumatic disease: a systematic review of meta-analyses
Source: BMC Musculoskelet Disord. 2014 Apr 7;15:121. doi: 10.1186/1471-2474-15-121 (PMC4107718; doi:10.1186/1471-2474-15-121)
Supplement: Additional file 1 — Search strategies for databases searched. [file 1471-2474-15-121-S1.docx]

**Additional File 1.** Search strategies for databases searched.

PubMed

Sport Discus

Web of Science

Scopus

ProQuest

Cochrane Database of Systematic Reviews (CDSR)

Physiotherapy Evidence Database (PEDRO)

Database of Abstract of Reviews of Effects (DARE)

Health Evidence Canada (HEC)
