# Supplementary material for: Effects of exercise on depressive symptoms in adults with arthritis and other rheumatic disease: a systematic review of meta-analyses
Source: BMC Musculoskelet Disord. 2014 Apr 7;15:121. doi: 10.1186/1471-2474-15-121 (PMC4107718; doi:10.1186/1471-2474-15-121)
Supplement: Additional file 2 — Studies excluded, including reasons for exclusion. [file 1471-2474-15-121-S2.docx]

**Additional File 2.** Studies excluded, including reasons for exclusion.

(1) Bariatric surgery for people with diabetes and morbid obesity: an evidence-based analysis. Ontario Health Technolology Assessment Services 2009;9(22):1-23. Inappropriate Population, Inappropriate Intervention

(2) Management of fibromyalgia. Drug and Therapeutics Bulletin 2010;48(8):89-93. Inappropriate Study Design

(3) Adler PA. The effects of Tai Chi on pain and function in older adults with osteoarthritis. Cleveland. Case Western Reserve University; 2007. Inappropriate Study Design

(4) Allen LA, Escobar J, I, Lehrer PM, Gara MA, Woolfolk RL. Psychosocial treatments for multiple unexplained physical symptoms: a review of the literature. Psychosomatic Medicine 2002;64(6):939-50. Inappropriate Population

(5) Barlow J, Singh D, Bayer S, Curry R. A systematic review of the benefits of home telecare for frail elderly people and those with long-term conditions. Journal of Telemedicine and Telecare 2007;13(4):172-9. Inappropriate Study Design, Inappropriate Intervention

(6) Beltran R. The effects of a supervised group aerobic exercise program and a chronobiologically oriented treatment protocol on symptomatology and mood in women with fibromyalgia. Alliant International University, San Diego; 2003. Inappropriate Study Design

(7) Bernardy K, Fuber N, Kollner V, Hauser W. Efficacy of cognitive-behavioral therapies in fibromyalgia syndrome: a systematic review and metaanalysis of randomized controlled trials. Journal of Rheumatology 2010;37(10):1991-2005. Inappropriate Intervention, Inappropriate Comparison Group

(8) Berzins K, Reilly S, Abell J, Hughes J, Challis D. UK self-care support initiatives for older patients with long-term conditions: a review. Chronic Illness 2009;5(1):56-72. Inappropriate Population

(9) Borkhoff CM, Wieland ML, Myasoedova E, Ahmad Z, Welch V, Hawker GA, Li LC, Buchbinder R, Ueffing E, Beaton D, Cardiel MH, Gabriel SE, Guillemin F, Adebajo AO, Bombardier C, Hajjaj-Hassouni N, Tugwell P. Reaching those most in need: a scoping review of interventions to improve health care quality for disadvantaged populations with osteoarthritis. Arthritis Care and Research 2011;63(1):39-52. Inappropriate Study Design, Inappropriate Outcomes

(10) Brosseau, L., Wells, G. A., Tugwell, P., Egan, M., Wilson, K. G., Dubouloz, C. J., Casimiro, L., Robinson, V. A., McGowan, J., Busch, A., Poitras, S., Moldofsky, H., Harth, M., Finestone, H. M., Nielson, W., Haines-Wangda, A., Doreleyers, M. R., Lambert, K., Marshall, A. D., Veilleux, L., Busch, A., Poitras, S., Moldofsky, H., Harth, M., Finestone, H. M., Nielson, W., Haines-Wangda, A., Russel-Doreleyers, M., Lambert, K., Marshall, A. D., and Veilleux, L. Ottawa Panel evidence-based clinical practice guidelines for aerobic fitness exercises in the management of fibromyalgia: Part 1. Physical Therapy 88(7), 857-871. 2008. Inappropriate Study Design

(11) Brosseau, L., Wells, G. A., Tugwell, P., Egan, M., Wilson, K. G., Dubouloz, C. J., Casimiro, L., Robinson, V. A., McGowan, J., Busch, A., Poitras, S., Moldofsky, H., Harth, M., Finestone, H. M., Nielson, W., Haines-Wangda, A., Russell-Doreleyers, M., Lambert, K., Marshall, A. D., and Veilleux, L. Ottawa Panel evidence-based clinical practice guidelines for strengthening exercises in the management of fibromyalgia: Part 2. Physical Therapy 88(7), 873-886. 2008. Inappropriate Study Design

(12) Calandre EP, Rico-Villademoros F. The role of antipsychotics in the management of fibromyalgia. CNS Drugs 2012 February 1;26(2):135-53. Inappropriate Study Design, Inappropriate Intervention

(13) Cancelliere C. Are workplace health promotion/wellness programs effective at improving presenteeism (on-the-job productivity) in workers? A systematic review and best evidence synthesis of the literature. Lakehead University (Canada); 2011. Inappropriate Study Design, Inappropriate Population, Inappropriate Outcomes

(14) Carnes D, Homer KE, Miles CL, Pincus T, Underwood M, Rahman A, Taylor SJ. Effective delivery styles and content for self-management interventions for chronic musculoskeletal pain: a systematic literature review. Clinical Journal of Pain 2012;28(4):344-54. Inappropriate Population

(15) Carville SF, Choy EHS. Systematic review of discriminating power of outcome measures used in clinical trials of fibromyalgia. Journal of Rheumatology 2008;35(11):2094-105. Inappropriate Intervention, Inappropriate Comparison Group

(16) Carville SF, Arendt-Nielsen S, Bliddal H, Blotman F, Branco JC, Buskila D, Da Silva JAP, Danneskiold-Samsoe B, Dincer F, Henriksson C, Henriksson KG, Kosek E, Longley K, McCarthy GM, Perrot S, Puszczewicz M, Sarzi-Puttini P, Silman A, Spath M, Choy EH. EULAR evidence-based recommendations for the management of fibromyalgia syndrome. Annals of the Rheumatic Diseases 2008;67(4):536-41. Inappropriate Study Design

(17) Cazzola M, Atzeni F, Salaffi F, Stisi S, Cassisi G, Sarzi-Puttini P. Which kind of exercise is best in fibromyalgia therapeutic programmes? A practical review. Clinical and Experimental Rheumatology 2010 November;28(6 Suppl 63):S117-S124. Inappropriate Study Design

(18) Centre for Reviews and Dissemination. Systematic review of the clinical effectiveness of self care support networks in health and social care. Report. 2006. Inappropriate Intervention, Inappropriate Study Design,

(19) D'Silva S, Poscablo C, Habousha R, Kogan M, Kligler B. Mind-body medicine therapies for a range of depression severity: A systematic review. Psychosomatics 2012;53(5):407-23. Inappropriate Study Design

(20) Dagfinrud H, Halvorsen S, Vollestad NK, Niedermann K, Kvien TK, Hagen KB. Exercise programs in trials for patients with ankylosing spondylitis: Do they really have the potential for effectiveness? Arthritis Care and Research 2011;63(4):597-603. Inappropriate Study Design, Inappropriate Outcomes

(21) Epstein I, Stinson J, Stevens B. The effects of camp on health-related quality of life in children with chronic illnesses: a review of the literature. Journal of Pediatric Oncology Nursing 2005;22(2):89-103. Inappropriate Population, Inappropriate Study Design

(22) Garcia-Campayo J, Magdalena J, Magallon R, Fernandez-Garcia E, Salas M, Andres E. A meta-analysis of the efficacy of fibromyalgia treatment according to level of care. Arthritis Research and Therapy 2008;10(4). Inappropriate Intervention, Inappropriate Comparison Group

(23) Gellis ZD, Kang-Yi C. Meta-analysis of the effect of cardiac rehabilitation interventions on depression outcomes in adults 64 years of age and older. American Journal of Cardiology 2012 November 1;110(9):1219-24. Inappropriate Population

(24) Glombiewski JA, Sawyer AT, Gutermann J, Koenig K, Rief W, Hofmann SG. Psychological treatments for fibromyalgia: A meta-analysis. Pain 2010;151(2):280-95. Inappropriate Intervention

(25) Grossman P, Niemann L, Schmidt S, Walach H. Mindfulness-based stress reduction and health benefits: a meta-analysis. Journal of Psychosomatic Research 2004;57(1):35-43. Inappropriate Outcomes

(26) Hadhazy VA, Ezzo J, Creamer P, Berman BM. Mind-body therapies for the treatment of fibromyalgia: a systematic review. Journal of Rheumatology 2000;27(12):2911-8. Inappropriate Study Design

(27) Han TS, Tajar A, Lean ME. Obesity and weight management in the elderly. British Medical Bulletin 2011;97:169-96. Inappropriate Population, Inappropriate Outcomes

(28) Hauser W, Bernardy K, Arnold B, Offenbacher M, Schiltenwolf M. Efficacy of multicomponent treatment in fibromyalgia syndrome: A meta-analysis of randomized controlled clinical trials. Arthritis Care and Research 2009;61(2):216-24. Inappropriate Intervention, Inappropriate Comparison Group

(29) Hauser W, Thieme K, Turk DC. Guidelines on the management of fibromyalgia syndrome - A systematic review. European Journal of Pain 2010;14(1):5-10. Inappropriate Study Design

(30) Herring MP, Puetz TW, O'Connor PJ, Dishman RK. Effect of exercise training on depressive symptoms among patients with a chronic illness: A systematic review and meta-analysis of randomized controlled trials. Archives of Internal Medicine 2012;172 (2):101-111. Inappropriate Population

(31) Holdcraft LC, Assefi N, Buchwald D. Complementary and alternative medicine in fibromyalgia and related syndromes. Best Practice and Research in Clinical Rheumatology 2003;17(4):667-83. Inappropriate Study Design, Inappropriate Intervention

(32) Imamura M, Cassius DA, Fregni F. Fibromyalgia: From treatment to rehabilitation. European Journal of Pain 2009 November 1;3(2):117-22. Inappropriate Study Design

(33) Jones KD, Deodhar P, Lorentzen A, Bennett RM, Deodhar AA. Growth hormone perturbations in fibromyalgia: a review. Semininars in Arthritis and Rheumatism 2007 June;36(6):357-79. Inappropriate Intervention, Inappropriate Outcomes

(34) Karmisholt K, Gotzcshe PC. Physical activity for secondary prevention of disease - Systematic reviews of randomised clinical trials. Danish Medical Bulletin 2005;52(2):90-4. Inappropriate Study Design

(35) Keysor JJ, Jette AM. Have we oversold the benefit of late-life exercise? Journals of Gerontology Series A - Biological Sciences and Medical Sciences 2001;56A(7):M412-M423. Inappropriate Study Design, Inappropriate Population

(36) Kiepe MS, Stockigt B, Keil T. Effects of dance therapy and ballroom dances on physical and mental illnesses: A systematic review. Arts in Psychotherapy 2012;39(5):404-11. Inappropriate Study Design

(37) Knittle K, Maes S, de G, V. Psychological interventions for rheumatoid arthritis: examining the role of self-regulation with a systematic review and meta-analysis of randomized controlled trials. Arthritis Care and Research 2010 October;62(10):1460-72. Inappropriate Intervention, Inappropriate Comparison Group

(38) Kovar PA. Impact of a supervised walking and education program on functional status: Results from a randomized controlled trial in patients with osteoarthritis of the knee. Columbia University Teachers College; 1991. Inappropriate Study Design

(39) Kroenke K. Efficacy of treatment for somatoform disorders: a review of randomized controlled trials. Psychosomatic Medicine 2007;69(9):881-8. Inappropriate Study Design, Inappropriate Population

(40) La MK, Marks R. The efficacy of aerobic exercises for treating osteoarthritis of the knee. New Zealand Journal of Physiotherapy 1995;23(2):23-30. Inappropriate Study Design

(41) Lange AK, Vanwanseele B, Fiatarone Singh MA. Strength training for treatment of osteoarthritis of the knee: a systematic review. Arthritis and Rheumatism (Arthritis Care and Research) 2008 October 15;59(10):1488-94. Inappropriate Study Design

(42) Langhorst J, Klose P, Dobos GJ, Bernardy K, Hauser W. Efficacy and safety of meditative movement therapies in fibromyalgia syndrome: a systematic review and meta-analysis of randomized controlled trials. Rheumatology International 2013;33 (1):193-207. Inappropriate Population

(43) Lee MS, Pittler MH, Ernst E. Tai chi for rheumatoid arthritis: systematic review. Rheumatology 2007 November;46(11):1648-51. Inappropriate Study Design

(44) Lima, T. B., Dias, J. M., Mazuquin, B. F., da Silva, C. T., Nogueira, R. M. P., Marques, A. P., Lavado, E. L., and Cardoso, J. R. The effectiveness of aquatic physical therapy in the treatment of fibromyalgia: a systematic review with meta-analysis. Clinical Rehabilitation 27(10), 892-908. 2013.

(45) Luciano JV, Martinez N, Penarrubia-Maria MT, Fernandez-Vergel R, Garcia-Campayo J, Verduras C, Blanco ME, Jimenez M, Ruiz JM, del Hoyo YL, Serrano-Blanco A. Effectiveness of a psychoeducational treatment program implemented in general practice for fibromyalgia patients: A randomized controlled trial. Clinical Journal of Pain 2011;27(5):383-91. Inappropriate Study Design

(46) Mannerkorpi K, Iversen MD. Physical exercise in fibromyalgia and related syndromes. Best Practice and Research: Clinical Rheumatology 2003;17(4):629-47. Inappropriate Study Design

(47) Mansky P, Sannes T, Wallerstedt D, Ge A, Ryan M, Johnson LL, Chesney M, Gerber L. Tai chi chuan: mind-body practice or exercise intervention? Studying the benefit for cancer survivors. Integrative Cancer Therapies 2006;5(3):192-201. Inappropriate Study Design, Inappropriate Population

(48) McIlvane JM, Baker TA, Mingo CA, Haley WE. Are behavioral interventions for arthritis effective with minorities? Addressing racial and ethnic diversity in disability and rehabilitation. Arthritis and Rheumatism (Arthritis Care and Research) 2008;59(10):1512-8. Inappropriate Study Design, Inappropriate Population

(49) McVeigh JG, McGaughey H, Hall M, Kane P. The effectiveness of hydrotherapy in the management of fibromyalgia syndrome: a systematic review. Rheumatology International 2008;29(2):119-30. Inappropriate Study Design, Inappropriate Outcomes

(50) Minor MA. Exercise maintenance behavior of subjects with arthritis following participation in a supervised exercise program. University of Missouri - Columbia; 1989. Inappropriate Study Design

(51) Onubogu U. Relationships among pain, pain medication, and pain outcomes in older adults with arthritis. Ann Arbor: University of Florida; 2008. Inappropriate Study Design, Inappropriate Intervention

(52) Pariser DA. The effects of telephone intervention on arthritis self-efficacy, depression, pain, and fatigue in older adults with arthritis. University of New Orleans; 2004. Inappropriate Study Design, Inappropriate Intervention

(53) Perraton L, Machotka Z, Kumar S. Components of effective randomized controlled trials of hydrotherapy programs for fibromyalgia syndrome: a systematic review. Journal of Pain Research 2009;2:165-73. Inappropriate Study Design

(54) Ross SD, Estok RP, Frame D, Stone LR, Ludensky V, Levine CB. Disability and chronic fatigue syndrome - A focus on function. Archives of Internal Medicine 2004;164(10):1098-107. Inappropriate Study Design, Inappropriate Population

(55) Somers TJ, Keefe FJ, Godiwala N, Hoyler GH. Psychosocial factors and the pain experience of osteoarthritis patients: new findings and new directions. Current Opinion in Rheumatology 2009 September;21(5):501-6. Inappropriate Study Design, Inappropriate Outcomes

(56) Stetter F, Kupper S. Autogenic training: a meta-analysis of clinical outcome studies. Applied Psychophysiology and Biofeedback 2002;27(1):45-98. Inappropriate Intervention

(57) Strombeck B, Jacobsson LT. The role of exercise in the rehabilitation of patients with systemic lupus erythematosus and patients with primary Sjogren's syndrome. Current Opinion in Rheumatology 2007 March;19(2):197-203. Inappropriate Study Design

(58) Thomas EN, Blotman F. Aerobic exercise in fibromyalgia: a practical review. Rheumatology International 2010 July;30(9):1143-50. Inappropriate Study Design

(59) Tschentscher M, Niederseer D, Niebauer J. Health benefits of nordic walking: A systematic review. American Journal of Preventive Medicine 2013 January 1;44(1):76-84. Inappropriate Study Design, Inappropriate Population

(60) Vincent KR, Vincent HK. Resistance exercise for knee osteoarthritis. Physical Medicine and Rehabilitation 2012 May;4(5 Suppl):S45-S52. Inappropriate Study Design

(61) Voigt-Radloff S, Schochat T, Heiss HW. Controlled trials on the efficacy of occupational therapy with elderly. Part II: Evidence for prioritised diseases and disabilities. Zeitschrift fur Gerontologie und Geriatrie 2004;37(6):450-8. Inappropriate Study Design

(62) Wang C, Collett JP, Lau J. The effect of Tai Chi on health outcomes in patients with chronic conditions: a systematic review. Archives of Internal Medicine 2004;164(5):493-501. Inappropriate Intervention

(63) Wang WC, Zhang AL, Rasmussen B, Lin LW, Dunning T, Kang SW, Park BJ, Lo SK. The effect of Tai Chi on psychosocial well-being: a systematic review of randomized controlled trials. Journal of Acupuncture and Meridian Studies 2009;2(3):171-81. Inappropriate Population

(64) Wei CY. Effects of topical 024(TM) essential oils during a 12-week exercise program on functional health and well-being of women with fibromyalgia syndrome. California State University, Fullerton; 2006. Inappropriate Study Design, Inappropriate Comparison Group

(65) Winbush NY, Gross CR, Kreitzer MJ. The effects of mindfulness-based stress reduction on sleep disturbance: a systematic review. Explore: Journal of Science and Healing 2007;3(6):585-91. Inappropriate Population, Inappropriate Outcomes

(66) Yohannes AM, Caton S. Management of depression in older people with osteoarthritis: A systematic review. Aging and Mental Health 2010;14(6):637-51. Inappropriate Study Design

(67) Zhang J, Wei W, Wang CM. Effects of psychological interventions for patients with systemic lupus erythematosus: a systematic review and meta-analysis. Lupus 2012 September;21(10):1077-87. Inappropriate Intervention
